# Development of sexual dimorphism in two sympatric skinks with different growth rates

**DOI:** 10.1002/ece3.5358

**Published:** 2019-06-14

**Authors:** Chen Yang, Jinming Zhao, Raul E. Diaz, Nan Lyu

**Affiliations:** ^1^ Chengdu Institute of Biology Chinese Academy of Sciences Chengdu China; ^2^ Key Laboratory of Southwest China Wildlife Resources Conservation (Ministry of Education) China West Normal University Nanchong China; ^3^ School of Resources and Environmental Engineering Anhui University Hefei China; ^4^ Department of Biological Sciences Southeastern Louisiana University Hammond Louisiana; ^5^ Ministry of Education Key Laboratory for Biodiversity and Ecological Engineering College of Life Sciences Beijing Normal University Beijing China

**Keywords:** growth curves, model, sexual size dimorphism, skink, sympatric, trade‐offs

## Abstract

Sexual size dimorphism (SSD) is widespread in animals, especially in lizards (Reptilia: Squamata), and is driven by fecundity selection, male–male competition, or other adaptive hypotheses. However, these selective pressures may vary through different life history periods; thus, it is essential to assess the relationship between growth and SSD. In this study, we tracked SSD dynamics between a “fading‐tail color skink” (blue tail skink whose tail is only blue during its juvenile stage: *Plestiodon elegans*) and a “nonfade color” tail skink (retains a blue tail throughout life: *Plestiodon quadrilineatus*) under a controlled experimental environment. We fitted growth curves of morphological traits (body mass, SVL, and TL) using three growth models (Logistic, Gompertz, and von Bertalanffy). We found that both skinks have male‐biased SSD as adults. Body mass has a higher goodness of fit (as represented by very high *R* ^2^ values) using the von Bertalanffy model than the other two models. In contrast, SVL and TL for both skinks had higher goodness of fit when using the Gompertz model. Two lizards displayed divergent life history tactics: *P. elegans* grows faster, matures earlier (at 65 weeks), and presents an allometric growth rate, whereas *P. quadrilineatus* grows slower, matures later (at 106 weeks), and presents an isometric growth rate. Our findings imply that species‐ and sex‐specific trade‐offs in the allocation of energy to growth and reproduction may cause the growth patterns to diverge, ultimately resulting in the dissimilar patterns of SSD.

## 1 INTRODUCTION

Sexual size dimorphism (SSD) is a widespread and fundamental biological phenomenon in which one sex is characteristically larger than the opposite sex. SSD is usually characterized by some morphological traits being more developed or exaggerated in one particular sex (Shine, 1989), but SSD may also act differentially on various morphological traits, causing dimorphism in trait size or in shape independent of size (ornamentation, scalation, or coloration; Schwarzkopf, 2005). The evolutionary basis of such dimorphic phenotypes has usually been associated with fitness and resource availability for each sex. Several hypotheses have been proposed to explain the evolutionary causes for SSD. The male–male competition hypothesis suggests that males become larger than females due to male–male competition for female mates or are directly chosen for their size by females (Gienger & Beck, 2007; Ji, Lin, Lin, Qiu, & Du, 2006). In contrast, the fecundity advantage hypothesis proposes that larger females present a larger body trunk volume to carry more eggs or produce larger eggs to increase offspring survival (Braña, 1996; Cox, Skelly, & John‐Alder, 2003; Scharf & Meiri, 2013). Thus, sexual selection favors larger males, whereas fecundity selection favors larger females. A third alternative is the intersexual competition hypothesis, in which both sexes avoid intersexual competition due to sex‐specific adaptations to different ecological niches (Blanckenhorn, 2005). In this case, ecological traits, including dietary divergence, habitat requirements, growth rates, growth strategies, population density, geographic variation, and disease, may drive the evolution of SSD (Hierlihy, Garcia‐Collazo, B. Chavez Tapia, & Mallory, 2013; Isaac, 2005; Stamps & Krishnan, 1997; Stamps, Losos, & Andrews, 1997; Zhong, Liu, Li, Peng, & Guo, 2017). Moreover, some endogenous mechanisms such as hormone regulation could also induce SSD in some animal groups (Kubička, Golinski, John‐Alder, & Kratochvíl, 2013). These hypotheses are not mutually exclusive; SSD may evolve under a combination of fecundity selection, male–male competition, and natural selection (Isaac, 2005).

Typically, while assessing the selection on sex dimorphic traits, whole‐body size measurements are recorded but selection acting on specific body parts, such as the head, tail, teeth, or limbs, can also induce size dimorphism (Braña, 1996; Bülbül, Kurnaz, Eroğlu, Koç, & Kutrup, 2016), and this is often overlooked. In addition, many single‐species studies focus on adult dimorphic traits and their corresponding fitness consequences. However, individuals may face changing selections during different life history periods (Cooper & Vitt, 1985); therefore, assessing the development of SSD during different life history periods may play important roles in unraveling the evolution of SSD.

Lizards show considerable variation in both direction and magnitude of SSD and have long been used as model species in SSD studies (Bonneaud et al., 2016; Lande, 1980; Manicom, Alford, Schoener, & Schwarzkopf, 2014; Shine, 1989). There are 50 currently recognized species of skinks in genus *Plestiodon*, which are widely distributed in the Palearctic, Oriental, and Nearctic realm (Brandley et al., 2012). The young of many species of skinks present a conspicuous brilliant blue tail that may be used to distract the attention of predators, because the tail can be autotomized to allow the individual an opportunity to escape (Cooper & Vitt, 1985; Uetz, Freed, & Jirí, 2018). This blue tail coloration fades with age and, for species belonging to the genus *Plestiodon*, is lost at sexual maturation (Hawlena, Boochnik, Abramsky, & Bouskila, 2006; Vitt & Cooper, 1986). However, the Chinese four‐lined skink, *Plestiodon quadrilineatus*, retains the blue tail into adulthood postsexual maturity (Lazell & Ota, 2000; Zhao, Zhao, & Zhou, 1999). Why congeneric and sympatric skinks adopt different life history tactics? It may be related to specific ontogenic process on two different species: Fading blue tail skinks accelerate growth into adulthood followed by a continued growth at a slower rate to enable earlier breeding, while nonfade blue tail skinks grow slowly and mature later. We suggest that differential ontogenetic color change of their blue tails may ultimately play a role in generating divergent patterns of SSD in these two skinks. Previous research in mammalian populations has indicated that dynamic growth patterns of individuals can shape variation in life history traits (Lammers, Dziech, & German, 2001). However, the growth patterns and processes that lead to SSD in reptiles are not well quantified. Heterochrony analysis could be used to examine differences in growth rates and timing of developmental events between different lizard species.

In order to test our above hypothesis, we selected two sympatric skink species—the faded tail skink (Blue tail skink: *Plestiodon elegans*) to serve as a comparison with *P. quadrilineatus* and reared individuals of each species in a controlled laboratory study—since factors such as food availability, thermal conditions, and predator risk may affect the longevity and life history traits significantly in lizards, including growth rate (Bonneaud et al., 2016; Kaliontzopoulou, Carretero, & Adams, 2015). We then collected different morphological data from the two skink species and fitted the growth curves of both sexes and traced the SSD in morphological characteristics between the two species. Our objectives were as follows: (a) to explore the morphological growth processes between two sympatric skink species; (b) to compare species‐ and sex differences in size and shape of the morphological traits (i.e., the patterns of SSD); and (c) to assess the development of SSD and the relationships with growth rate for each morphological trait. Based on the knowledge of sex‐ and species‐specific growth trajectories, we present an improved understanding and interpretation of the ontogenetic pattern of sexual dimorphism, and ultimately for understanding the evolutionary significance of life history trait differences in genus *Plestiodon*.

## 2 MATERIALS AND METHODS

### 2.1 Sampling animal collection

**Figure 1:**
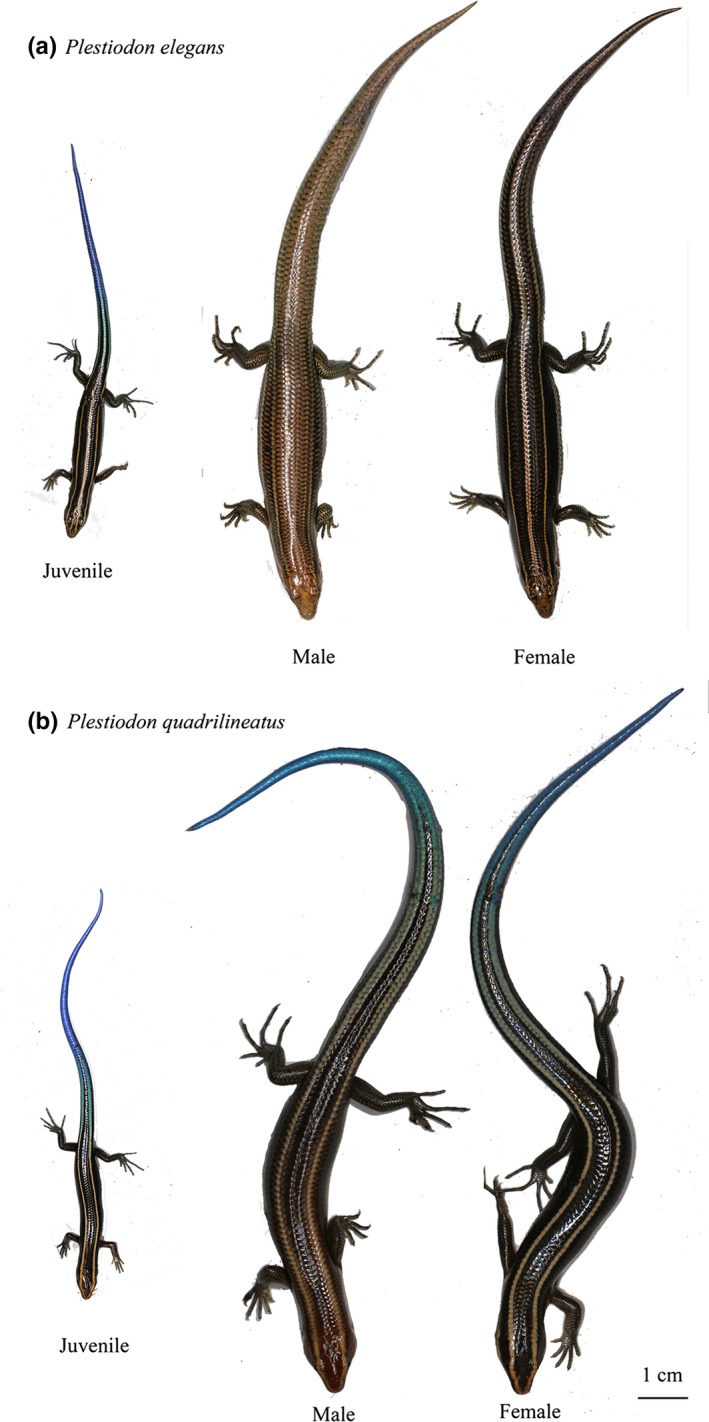
Photographs of Juvenile, female, and male adults of both Skinks. (a) *Plestiodon elegan*s: Mature males have slightly reddish brown color on the ventral surface of the trunk, whereas mature females are blackish brown. (b) *Plestiodon quadrilineatus*: Mature males have relatively brownish red and broader heads than females

Individuals for both skink species (weighing approximately 1.4 g) were collected from the Xiaotao Skink Farm at Xin'an County, Guangxi Province, China (110.68°E, 25.61°N), in March 2016 (Figure 1) for a total *P. elegans* (males = 13, females = 11) and *P. quadrilineatus* (males = 9, females = 7). All skinks were collected 4 weeks after hatching at the skink farm. We first placed individuals in a plastic box filled with grasses and foods (insects) and were then transferred to our laboratory facilities at the Chengdu Institute of Biology (CIB), Chinese Academy of Sciences (CAS; 104.06°E, 30.67°N). No skinks perished during transportation. All experimental procedures on animals used in the present study were approved by the Animal Care and Use Committee of the CIB, CAS. Permits for animal collection and observation were approved by the Department of Wildlife Management, Bureau of Gansu Forestry Administration, Sichuan province, China. All staff, fellows, and students received appropriate training before performing the laboratory studies.

### 2.2 Animal husbandry

In the laboratory, all skinks were housed in standard plastic cages (26 cm L × 17.5 cm W × 12.5 cm H). To avoid mutual interference and fighting, only one skink was placed in each cage. All cages were filled with 3‐cm‐deep substrate of coconut soil (Nomoypet Products) and 2–3 cm cured hay to cover the soil and provide a layer for the skink to burrow beneath and aid in thermoregulation. A single 3‐cm‐diameter × 1‐cm‐high plastic box was filled with tap water and placed at the corner of each cage, and five grams of nutrient powder was mixed in the water each week. The powder included vitamin A, vitamin D, and various trace minerals (Repti Calcium®; Nomoypet Products). Yellow mealworm (*Tenebrio molitor*), Needle crickets (*Achetus domesticus*), and Turkistan Roaches (*Blatta lateralis*) were provided as the primary foods once a week.

Experiments were conducted under a photoperiod of 12‐hr L (light):12‐hr D (dark), and illumination by an incandescent light bulb (about 70 lux) mounted on the roof was provided from 07:00 to 19:00. Without access to UV light, skink may suffer from metabolic bone disease due to vitamin D3‐related calcium deficiency (Adkins et al., 2003; Diaz et al., 2015). We also provided a UV lamp (Nomoypet: UVB 10.0) at the top of each cage from 12:00 to 14:00 using an automatic timer. Ambient room temperature was controlled at 24°C by the air‐conditioning system. To maintain constant cage temperatures, ceramic heat lamp (ReptileStructure Products, 100 W) was used at a distance of 20 cm to the cage, so that daytime thermal gradients in each cage ranged from 25 to 30°C (Shen, Pei, Lin, & Ji, 2017). In order to stimulate hibernation, the photoperiod was decreased to 6 hr per day and food was withheld during the first 2 weeks. During the following 2 weeks, no light source was provided and room temperature was controlled to a constant 15°C. Skinks were finally placed at a constant 8°C in a non‐air‐conditioned room for 8 weeks, where ambient temperature matched that found from their natural habitat. After 8 weeks, we provided the skinks with foods and nutrient powder, and maintained them under conditions identical to those under which they were reared.

### 2.3 Morphological trait measurements

All skinks were measured once a week following standard procedures (Zhao et al., 1999). Snout‐to‐vent length (SVL: from the anterior tip of the head to the cloacal opening) and tail length (TL: distance from vent to tip of tail) were measured with a ruler to the nearest 1 mm. Body mass was measured using digital electronic balance to the nearest 0.01 g. Sex could not be determined reliably for juveniles, until they grew to an adult or subadult size. Mature males were identified by their enlarged testes and convoluted epididymis typically associated with sperm production; and matured females contain vitellogenic follicles or oviducal eggs (Du & Ji, 2001; Lazell & Ota, 2000). In *P. elegans*, mature males have slightly reddish brown color on the ventral surface of the trunk, whereas mature females are blackish brown. In *P. quadrilineatus*, mature males have relatively brownish red and broader heads than the juveniles and females. After this experiment, we conducted gonadal inspection and found all the individuals at the time for 90% limit value for SVL were sexually mature, so we defined mature age at the time when the individual reached 90% limit value for SVL.

### 2.4 Statistical analysis

Continuous variables were tested for normality using Wilk–Shapiro normal tests. Trait measurements were ln‐transformed to meet the assumption of least‐squares regression and generalized linear model (GLM). We used independent *t* test to compare sexual traits between the two sexes and used GLM to test whether variation in sexual traits was explained by species. Because most variables varied between the two sexes, we controlled sex as a fixed effect in the models.

Growth curves provide a means for visualizing growth patterns over time, and the equations can be used to predict the mathematical expectations at a specific time. Three common models (logistic, Gompertz, and von Bertalanffy) were previously used in several other lizard studies (Guarino, Di Già, & Sindaco, 2010; Wapstra, Swain, & O'Reilly, 2001). When fitting the SVL growth curve models, SVL*t* is the average SVL (mm) at age *t* (weeks), *A* is the upper asymptotic or maximum SVL, *B* is a scaling parameter (constant of integration) which is established by the initial values, and *k* is the growth coefficient that the characteristic growth rate at which a skink approaches this size (shape of the growth curve) (Kaufmann, 1981).

Parameters of *A*,* B*, and *K* were estimated by *DUD* methods in *proc nlin* function of the SAS (Ralston & Jennrich, 1978). We first set BEST =* *10, which indicated the residual sums of squares only for the best 10 combinations of possible starting values from the grid. We next started to iterate the best model of these 10 combinations (with minimum sums of squares) using the Gauss–Newton method. Finally, we estimated the parameters of *A*,* B,* and *K* based on the iteration achieved at a minimum value. Parameters were calculated separately for males and females. To evaluate the goodness of fit of these models to the growth values, we use the *R* ^2^ value of the linear regression between observed and predicted growth to indicate the goodness of fit.

Absolute growth rate (AGR) is the instantaneous rate of growth estimated by the first derivative of the growth curve model. The differential equation for AGR is that $\mathrm{AGR}=\frac{dy}{dt}$; Relative growth rate (RGR) is the rate of growth divided by the size (*S*). *S* can be any measure such as TL, SVL, and body mass. RGR is expressed as the quotient of two differentials: $\mathrm{RGR}=\frac{1}{S}\times \frac{dy}{dt}$

For estimating the values of SVL, mass, and TL in adults, we first fitted the best growth model for each trait and then calculated, at the time for reaching the 90% limit values (LV) based on the best model, final recorded trait value for each individual (Tables 1 and 2). All results are shown as mean ± *SE*, and all tests were two‐tailed. Above statistical analyses were performed with SAS 9.2 (SAS Institute Inc., 2002. Version 9.2. SAS Institute, Cary, NC).

**Table 1:** Equation and parameter estimation of three different growth curve models

Models	Logistic	Gompertz	Von Bertalanffy
Equations	*Y* = *A*/(1 + *B*e^−*Kt*^)	*Y* = *A*e^−*B*exp(−*Kt*)^	*Y* = *A*(1 − *B*e^−*Kt*^)^3^
Absolute growth rate	*ABK*e^−*Kt*^/(1 + *B*e^−*Kt*^)^2^	*KAB*e^−*B*exp(−*Kt*)^e^−*Kt*^	3*KA*(1 − *B*e^−*Kt*^)^2^ *B*e^−*Kt*^
Relative growth rate	*K*(1 − *y*/*A*)	*K*(ln *A* − ln*yK*)	3*K*((*A*/*y*)^1/3^−1)
Upper asymptote	*A*	*A*	*A*
50% LV (weeks)	ln 5*B*/*K*	$\left(\text{ln}\frac{B}{\text{ln}\;2}\right)/K$	$\left(\text{ln}\frac{B}{0.2063}\right)/K$
70% LV (weeks)	ln 7*B*/*K*	$\left(\text{ln}\frac{B}{\text{ln}\;1.4286}\right)/K$	$\left(\text{ln}\frac{B}{0.1121}\right)/K$
90% LV (weeks)	ln 9*B*/*K*	$\left(\text{ln}\frac{B}{\text{ln}\;1.1111}\right)/K$	$\left(\text{ln}\frac{B}{0.0345}\right)/K$

Note

Parameters are as follows: *t* = growth time from birth (week); *A* = the upper limit that the reliability approaches asymptotically as *t*→∞, or the maximum reliability that can be attained; *B* = constant; *K* = growth coefficient (shape of the growth curve). 50% LV: time for reaching 50% limit value; 70% LV: time for reaching 70% limit value; 90% LV: time for reaching 90% limit value.

**Table 2:** Parameter estimation and goodness of fit for the best growth curve among three models (Logistic, Gompertz, and Von Bertalanffy)

Species	Sex	Variables	Models	Parameters			*F* test		Growth rate (week)		
				*A*	*B*	*K*	*F*	*R* ^2^	50% LV	70% LV	90% LV
*Plestiodon elegans*	♂	Body mass	Bertalanffy	16.1011	0.551	0.0388	27.384	0.965	25.320	41.041	71.404
		SVL	Gompertz	8.8921	1.3778	0.0394	16.245	0.942	17.437	34.300	65.250
		TL	Gompertz	12.2906	1.298	0.0447	13.831	0.933	14.034	28.898	56.179
		Body mass	Bertalanffy	15.7578	0.5503	0.0426	68.179	0.986	23.031	37.350	65.005
	♀	SVL	Gompertz	8.3623	1.1447	0.0416	12.187	0.924	12.059	28.031	57.344
		TL	Gompertz	11.1422	1.2336	0.0396	16.898	0.944	14.557	31.335	62.129
*Plestiodon quadrilineatus*	♂	Body mass	Bertalanffy	20.349	0.577	0.025	18.047	0.947	41.479	66.075	113.579
		SVL	Gompertz	12.2337	1.227	0.023	9.431	0.904	24.830	53.718	106.736
		TL	Gompertz	15.5821	1.5615	0.0257	10.855	0.916	31.602	57.454	104.903
	♀	Body mass	Bertalanffy	18.0618	0.558	0.024	34.177	0.971	41.460	66.875	115.962
		SVL	Gompertz	10.355	1.1588	0.0229	8.382	0.893	22.441	51.455	104.705
		TL	Gompertz	13.2846	1.236	0.023	7.895	0.888	25.148	54.035	107.054

Note

*A*,* B*, and *K* were estimated by methods of *DUD* in nonlinear regression.

Details of each isolate code (*A*,* B*,* K*, 50% LV, 70% LV, and 90% LV) are given in Table 1.

## 3 RESULTS

### 3.1 Differences in morphological traits

**Figure 2:**
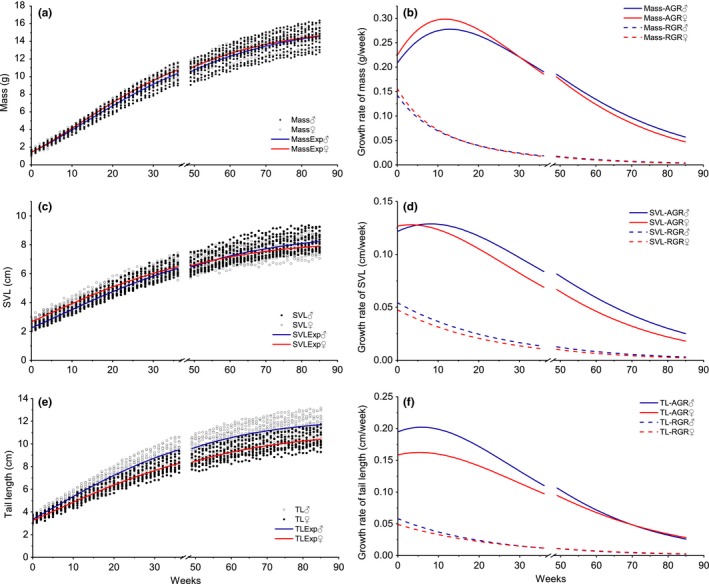
Growth rate and curve fitting on mass, SVL and TL for *Plestiodon elegans*. Parameters for these curves are presented in Table 2. AGR, absolute growth rate; Hibernation, from 37 to 48 weeks; MassExp, expected fitting curve for mass; MassTL, expected fitting curve for TL; RGR, relative growth rate; SVL, snout–vent length; SVLExp, expected fitting curve for SVL; TL, tail length

**Figure 3:**
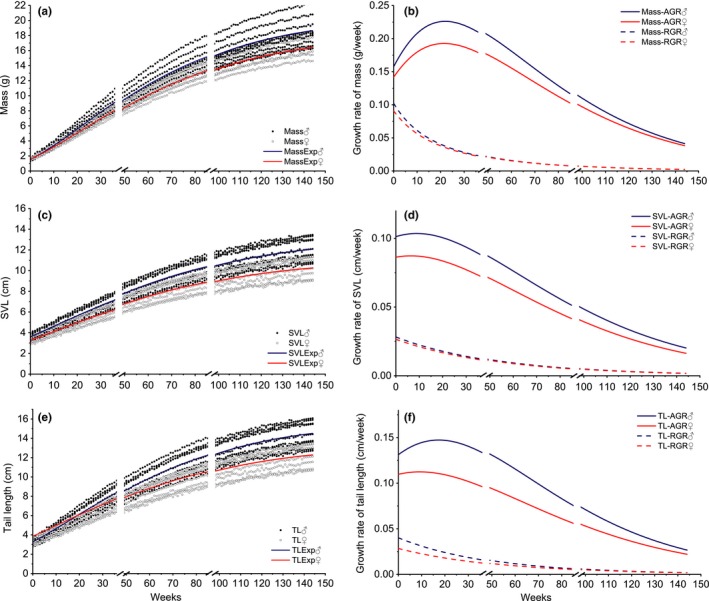
Growth rate and curve fitting on body mass, SVL and TL for *Plestiodon quadrilineatus*. Parameters for these curves are presented in Table 1. AGR, absolute growth rate; Hibernation, 37–48 weeks (break 1) and 86–97 weeks (break 2); MassExp, expected fitting curve for mass; MassTL, expected fitting curve for TL; RGR, relative growth rate; SVL, snout–vent length; SVLExp, expected fitting curve for SVL; TL, tail length

For hatchling *P. elegans*, there were no significant differences between males and females (independent *t* test: body mass: *t*_22_ = −1.065; *p* = 0.229, SVL: *t*_22_ = 0.506, *p *=* *0.618; TL: *t*_22_ = −1.171, *p *=* *0.254). Adult males (SVL: 8.61 ± 0.19 cm; TL: 12.15 ± 0.26 cm) were significantly larger than females (SVL: 7.60 ± 0.17 cm; TL: 10.95 ± 0.22 cm, *n *=* *11) (SVL: *t*_22_ = −3.914, *p *<* *0.05; TL: *t*_22_ = −3.431, *p *<* *0.05). No significant differences were found in body mass between males (15.55 ± 0.33 g, *n *=* *13) and females (14.83 ± 1.87 g, *n *=* *11) (body mass: *t*_22_ = −1.795, *p *=* *0.086). Similar for *P. quadrilineatus*, there were no significant differences in body mass between the two sexes (*t*_14_ = 0.118, *p *=* *0.908), SVL (*t*_14_ = −1.951, *p *=* *0.071), and TL (*t*_14_ = −2.041, *p *=* *0.061), but adult males of *P. quadrilineatus* attained larger sizes and greater weights (SVL: 11.22 ± 0.40 cm; TL: 14.85 ± 0.57 cm; body mass = 19.02 ± 0.17 g) than adult females (SVL: 9.75 ± 0.36 cm; TL: 12.47 ± 0.49 cm; body mass = 16.98 ± 0.27 g) (SVL: *t*_14_ = −2.952, *p *<* *0.05; TL: *t*_14_ = −3.089, *p *<* *0.05; body mass: *t*_14_ = −7.243, *p *<* *0.01). Adult *P. quadrilineatus* were larger in body size and weight than adult *P. elegans* (Figures 2 and 3). After controlling for sex, the significant difference on body mass can also be explained by species (GLM: body mass: *F*_1,40_ = 104.703, *p *<* *0.01; SVL: *F*_1,40_ = 85.974, *p *<* *0.01; TL: *F*_1,40_ = 30.919, *p *<* *0.01).

### 3.2 Model performance on growth circle and growth rate

Three models of Gompertz, Logistic, and von Bertalanffy were used to fit growth curves for both skink species (Table 1). Nonlinear regression showed that the *p* values for all these models were <0.05, suggesting that all three models were suitable to fit the growth curves (Table 2). However, goodness‐of‐fit tests for body mass, SVL, and tail length varied among models. Body mass model goodness of fit was better using the von Bertalanffy model as evident by higher *R* ^2^ values than other models (*R* ^2^ = 0.965 for male *P. elegans*,* R* ^2^ = 0.986 for female *P. elegans*, and *R* ^2^ = 0.947 for male *P. quadrilineatus*,* R* ^2^ = 0.971 for female *P. quadrilineatus*). However, goodness of fit for SVL and TL for both skinks was better using Gompertz model (Table 2). Estimations of growth of body mass, SVL, and TL for each individual based on the time required to complete growth revealed that male *P. elegans* reached 90% LV for TL over a shorter time period than females (56.179 vs. 62.129 weeks). Female *P. elegans* reached 90% LV for SVL and body mass in less time than males (body mass: 65.005 weeks; SVL: 57.344 weeks). We also found that the growth rate of TL for males was greater than in female *P. elegans* in an earlier period of the growth circle (Figure 2d,f). In contrast, females had a higher growth rate in body mass than males in the beginning growth period (Figure 2a,b). Generally, *P. quadrilineatus* presented slower growth and more delayed maturity than *P. elegans* (Table 2 and Figure 2). In *P. quadrilineatus*, the time for reaching 90% LV of body mass, SVL, and TL was all substantially over 100 weeks (Table 2). Growth rates for body mass, SVL, and TL were consistently higher in males than females (Figure 3b,d,f).

## 4 DISCUSSION

### 4.1 Development of SSD

In this study, we found no significant differences in body mass, SVL, and TL between hatchlings of the two sexes for both skink species. However, significant male‐biased SSD was identified in adults (Figures 2 and 3). It raises an important question that how would male‐biased SSD develop before adulthood. As sexes can differ markedly in the shape of their growth curves, overall rates of growth, duration of growth, and age at maturity, we assessed the development of SSD by considering all those factors that can themselves be subjected to selection. Specifically, adult *P. quadrilineatus* showed obvious male‐biased SSD in all three morphological traits (i.e., body mass, SVL, and TL), while *P. elegans* had similar body mass between both two sexes (but male‐biased SSD in SVL and TL). We suggest that this may be strongly related to their divergent life history strategies since *P. elegans* generally grows faster and matures earlier than *P. quadrilineatus* (Figures 2 and 3) which may also indicate that *P. elegans* can start to breed earlier than *P. quadrilineatus* (Lazell & Ota, 2000; Xu, Wu, & Wu, 2004). This may partially explain our findings on the growth patterns of different morphological traits. By calculating the growth rate, we have shown that under our laboratory conditions, female *P. elegans* had higher absolute growth rate on body mass than males during the early growth cycle, but male *P. elegans* had greater absolute growth rate at TL (Figure 2d,f). Thus, female *P. elegans* may prefer to invest more energy on body mass increasing to enable early breeding and to have relatively high fecundity (Du & Ji, 2001; Shu, Du, Zhao, & Hu, 2004). On the other hand, as male tail length has long been proved as an important mate choice criterion for female lizards (Gienger & Beck, 2007), male *P. elegans* may have to develop their tails in the first hand to match the relatively early breeding females (Cooper & Vitt, 1989; Huang, 1996). Previous laboratory research on other lizards also indicated that faster growth in certain body parts (e.g., development of wider jaws, larger SVL) could increase the probability of attracting a mate (Zhang, Tong, Wo, Liu, & Jin, 2018). Furthermore, *P. elegans* should face relatively strong selection due to the more limited energy intake during a shorter time period. Males and females have to trade‐off between developing certain body parts (e.g., tail) and increasing body mass which may cause divergent growth patterns for TL and body mass between the two sexes of *P. elegans* during the period immediately following hatchling. Also, if sexual selection on body mass is relatively weak, male *P. elegans* may benefit by maintaining a relatively light body (i.e., non‐SSD in body mass), which may allow them to be more highly mobile, and spend more time and energy on searching for mates instead of food (Trivers, 1976). This is typical for in populations where densities are low and females are widely dispersed, so that male mating success depends on the number of females encountered rather than on competitive advantages over other males (Zamudio, 1998).

The bright blue tail color functions to divert predatory attention away from the head and body, and the tail can be readily autotomized during predation events (Bateman, Fleming, & Rolek, 2014). Nonetheless, owning and maintaining a blue tail should be costly. On one hand, individuals would have to allocate more energy to produce the pigment materials to maintain the blue color. On the other hand, although they may have a higher probability of escaping from predations through autotomizing, tailless individuals have long been known to have impaired locomotor performance (Vitt & Cooper, 1986) and have to allocate additional energy to produce new tails. Therefore, the two skink species used in this study should apply two different strategies in tail color investment. *P. quadrilineatus* would keep the blue tail after sexual maturing, which may imply that they enjoy higher survivability by allocating more energy in maintaining the blue tails. In contrast, the blue tail color of *P. elegans* would gradually fade until adulthood, the trade‐off being that they must now save energy for growth and/or reproduction at the cost of facing higher predation risks. This is in line with our results that *P. elegans* grows faster and matures earlier than *P. quadrilineatus* (Figures 2 and 3) although we have no direct data to support the potential relationship between reduced mortality and delay at age of sexual maturity due to limited field studies on *P. quadrilineatus*. Presumably, longer time to mature enables the skinks to accept the costs from maintaining their blue tails, and as a trade‐off, they may have reduced predation.

### 4.2 Model fitting

Growth curves are empirical modes that can be used to study the evolution of a quantity over time, which is widely used in biology for quantities such as population size or biomass for population growth analysis (Werner & Gilliam, 1984; Westerbom, Kilpi, & Mustonen, 2002), and individual body size or biomass for growth analysis of individuals (Bjorndal, Parsons, Mustin, & Bolten, 2013; Halliday & Verrell, 1988). As useful tools to fit and predict growth tendencies, three classic models for growth curves (Gompertz, Logistic, and von Bertalanffy) were reported and applied in previous study (Kaufmann, 1981).

All the above models fitting the data are based on the method of nonlinear least squares and iterative procedures, so the related characteristic growth parameters (growth rate, inflection point, limiting value, etc.) can be derived from these fittings (Table 1). But these growth models were designed under a strong assumption that growth is a monotonically increasing function of time. Another condition in need of consideration is that many equations used to fit the growth curves are sensitive to irregularity of spacing of the size age points. Frequently, neither of the conditions could be held, especially for weight data collected under field conditions. Random events such as the stresses of climate, productivity, and diseases can also affect weight negatively and lead to a sharp decline from time to time. Moreover, growth parameters are often affected by different situations: temperature, food availability, population density, predator risk, etc. (Dunham, 1978; Schoener & Schoener, 1978; Sinervo, 1990), with similar situations arising in the experimental conditions (Duncan, Duncan, & Strycker, 2013).

Building on the previous studies that have used all three models of growth rates, all key conditions were controlled in our experiments to reduce the potential interference of such factors on ontogenetic growth and identify factors specific to sexes and between species. Furthermore, all morphological traits were measured weekly. To control for the effect of hibernation (no increment at this time), we added a break at the time for the curve fit (Figures 2 and 3). Our results showed that the best models for morphological fitting varied with morphological traits, but all three models for mass, SVL, and TL were high correlations for the goodness of fit (All *R* ^2^ > 0.85, *p *<* *0.05, Table 2), and these could be used for estimating age of both lizard species in future studies. Additionally, since we were mainly concerned about the growth pattern differences (but not the direct characteristic differences) between the two sympatric skinks in this study, we can effectively avoid the logical and statistical limitations of two‐species comparative studies given by Garland and Adolph (1994) since our comparisons were conducted for the same species under different life history periods.

## 5 CONCLUSIONS

Our findings support that male‐biased SSD in both skinks and fit the growth curves for morphological traits. The two species of skink display divergent life history strategies: *P. elegans* grows rapidly, matures early (at 65 weeks), and has allometric growth rate (TL), whereas *P. quadrilineatus* grows slowly, matures late (at 106 weeks), and has isogonic growth rate. Species‐ and sex‐specific trade‐offs in the allocation of energy to growth and reproduction cause the growth patterns to diverge, which may produce the dissimilar patterns of SSD. Our findings also highlight the importance for further researches to test the predictions of our hypothesis about the cause of the cline in sexual dimorphism of escape behavior and sexual selection, and to ascertain whether similar clines occur in other taxa.

## CONFLICT OF INTEREST

None declared.

## AUTHOR CONTRIBUTIONS

C.Y. and N.L. conceived the ideas and designed methodology; C.Y. and J.Z. collected the data; C.Y. and N.L. analyzed the data; C.Y., R.E.D., and N.L. led the writing of the manuscript. All authors contributed to drafts and gave final approval for publication.

## Data Availability

All the data generated for this study are uploaded to Dryad Digital Repository. DOI: https://doi.org/10.5061/dryad.nb25502.
